# Substance use and mental health in pediatric type 1 diabetes: key predictors of academic failure

**DOI:** 10.1192/j.eurpsy.2025.933

**Published:** 2025-08-26

**Authors:** C. Bey, T. Ach, A. Ben Abdelkarim

**Affiliations:** 1 University of Sousse, Faculty of Medicine of Sousse, 4000; 2 Endocrinology department, Farhat Hached University Hospital of Sousse, Sousse, Tunisia

## Abstract

**Introduction:**

Type 1 Diabetes (T1D) is an invisible condition, yet it can be difficult for young people to accept. Many attempt to hide or downplay their illness, potentially leading to significant psychological distress.

**Objectives:**

The objective of this study is to determine the prevalence and characteristics of academic failure among children with T1D, as well as the associated risk factors, including the impact of substance use such as tobacco, alcohol, and cannabis.

**Methods:**

This is a retrospective, descriptive, and analytical study conducted at the Endocrinology Department of Farhat Hached Hospital in Sousse, covering the period from January 2015 to January 2020. The study analyzed the school and professional trajectories of T1D patients, considering their clinical, biological, and social data. Academic failure, the dependent variable, was defined as either the interruption of studies or the repetition of at least one academic year. Additionally, the prevalence of smoking, alcohol, and cannabis use in this population was recorded.

**Results:**

The study included 70 patients (31 males and 39 females), with a mean age of diagnosis of T1D at 7.36±4.41 years. Academic repetition was observed in 71.4% of cases, and school dropout in 47.1%. The reasons for academic delays were attributed to recurrent hospitalizations (31.4%) and glycemic instability, including hyper/hypoglycemic episodes (17.1%). Multivariate analysis adjusted for diabetes type revealed that significant risk factors for academic failure included: ≥5 hospitalizations for ketosis (p=0.037) and higher mean HbA1c levels at recent consultations (p=0.001). Protective factors were functional insulin therapy (p=0.031) and the use of insulin analogs (p=0.004). Concerning substance use, tobacco addiction was present in 22.9% of patients, alcohol consumption in 14.3%, and cannabis use in 8.6%. Patients using tobacco and alcohol exhibited a higher risk of school dropout and lower academic performance, with a significant correlation between cannabis use and glycemic instability.

**Conclusions:**

The risk of academic failure among T1D patients is substantial and should not be underestimated. Frequent hospitalizations, poor glycemic control, and substance use (especially tobacco and cannabis) further exacerbate this risk. The physical and psychological complications of T1D, combined with the socioeconomic challenges and limited access to medical care in rural or isolated areas, contribute to school dropout. This study highlights the importance of comprehensive medical and psychosocial support to improve both health and academic outcomes in this vulnerable population.

**Disclosure of Interest:**

None Declared

